# Cellulose Nanocrystals Induced Loose and Porous Graphite Phase Carbon Nitride/Porous Carbon Composites for Capturing and Determining of Organochlorine Pesticides from Water and Fruit Juice by Solid-Phase Microextraction

**DOI:** 10.3390/polym15092218

**Published:** 2023-05-08

**Authors:** Huimin Li, Panlong Dong, Anying Long, Suling Feng, Jing Fan, Shengrui Xu

**Affiliations:** 1 Key Laboratory of Green Chemical Media and Reactions, Ministry of Education, Collaborative Innovation Center of Henan Province for Green Manufacturing of Fine Chemicals, School of Environment, School of Chemistry and Chemical Engineering, Henan Normal University, Xinxiang 453007, China; ming1391027849@163.com (H.L.); pldong2000@163.com (P.D.); slfeng@htu.cn (S.F.); fanjing@htu.cn (J.F.); 2 113 Geological Brigade, Bureau of Geology and Mineral Exploration and Development Guizhou Province, Liupanshui 553000, China

**Keywords:** cellulose nanocrystals, graphitized carbon nitride, organochlorine pesticides, solid-phase microextraction, gas chromatography–mass spectrometry

## Abstract

Herein, novel, loose, and porous graphite phase carbon nitride/porous carbon (g-C_3_N_4_@PC) composites were prepared by decorating cellulose nanocrystals (CNCs). The characterization results demonstrate that the as-prepared composites presented high specific surface areas, porous structures, and abundant chemical groups, with the modification of CNCs. In view of the unique advantages, g-C_3_N_4_@PC was used as the coating material for the solid-phase microextraction (SPME) of organochlorine pesticides (OCPs) in water and juice samples. The g-C_3_N_4_@PC-coated fibers showed better extraction efficiencies than commercial fibers (100/7 μm PDMS and PA) toward the OCPs, with the enrichment factors of the g-C_3_N_4_@PC-coated fibers 5–30 times higher than the latter. Using a gas chromatography–mass spectrometry (GC-MS) instrument, the g-C_3_N_4_@PC-coated fibers exhibited a gratifying analytical performance for determining low concentrations of OCPs, with a wide linear range (0.1–1600 ng L^−1^ for water; 0.1–1000 ng L^−1^ for juice), low limits of detection (0.0141–0.0942 ng L^−1^ for water; 0.0245–0.0777 ng L^−1^ for juice), and good reproducibility and repeatability in optimal conditions. The established method showed good sensitivity and recovery in the determination of OCPs in the water and fruit juice samples, which displayed broad prospects for analyzing organic pollutants from environmental samples.

## 1. Introduction

In the 1950s, organochlorine pesticides (OCPs) became prevalent [1] and were mainly used in agricultural production, significantly boosting the demand for food supply. However, OCPs have a high persistence and biomagnification effect [2]. On the one hand, they can accumulate in organisms for prolonged periods, and on the other hand, they can form the effect of long-distance migration through soil and surface water [3], ultimately endangering human health, causing various serious harmful diseases, such as cancer [4,5], reproductive dysfunction, birth defects, immunotoxicity, and neurotoxicity [6,7,8,9]. This is highly detrimental to human health and global ecological sustainable development. Due to its excessive harm, the use of organochlorine was banned in 2004. Although these OCPs have been banned, they may still remain in water samples [10], soils [11], sediments [12], aquatic organisms [13], and human bodies [14,15] due to their high persistence. Therefore, there is an urgent need to establish a sensitive and accurate analytical method to monitor OCP residues in the ecological environment, which is of great significance for human development.

In general, sample pretreatment is essential prior to the detection of OCPs using gas chromatography–mass spectrometry (GC-MS). Among various sample pretreatment techniques, solid-phase microextraction (SPME) attracted great interest owing to its unique advantages, such as solvent-free approach, high sensitivity, and straightforward operation [16,17,18]. Till now, SPME has been widely used in the analysis of environmental pollutants, food composition, drug content, biological components, etc. [19,20,21,22,23,24]. The extraction performance of SPME mainly depends on the coating materials, which determine the selectivity, sensitivity, and reproducibility of the analytical method [25,26,27]. Therefore, it is necessary to develop new SPME coating materials to meet diversified analysis for ultra-trace analytes.

As a new nonmetallic material, polymeric graphitized carbon nitride (g-C_3_N_4_) has attracted much attention owing to its advantages of a large specific surface area, excellent chemical and thermal stability, and low toxicity. Owing to these distinctive characteristics, g-C_3_N_4_ has be used as a new and efficient SPME coating material [28,29]. The two-dimensional nanosheet structure of g-C_3_N_4_ resembles that of graphene [30], and its layers are connected by conjugated π bonds formed by the sp^2^ hybridization of carbon and nitrogen atoms, as well as weak van der Waals forces [31,32,33]. Due to the incomplete condensation of the triazine ring and the existence of the delocalized π bond, g-C_3_N_4_ contains rich nitrogen-containing functional groups, electron-rich properties, and crystal defects, which provide abundant adsorption active sites for targeted compounds [34]. However, g-C_3_N_4_ tends to agglomerate during the synthesis process of thermal polymerization, which seriously affects its properties and leads to the reduction of adsorption sites [35]. Therefore, to overcome these limitations of g-C_3_N_4_, it is necessary to introduce a template material as the support material.

Cellulose nanocrystals (CNCs) possess numerous oxygen-containing functional groups, such as hydroxyl, on their surface, which aid in the formation of intermolecular interactions. At the same time, the hydrogen bonds formed by intermolecular interaction are conducive to the construction of three-dimensional structures [36]. Furthermore, the surface of CNCs contains a high concentration of hydroxyl groups that can be easily chemically modified [37]. Leveraging these advantages, CNCs can be used as the support material for g-C_3_N_4_.

In this study, g-C_3_N_4_ was prepared by calcining urea in a muffle furnace. Subsequently, graphite phase carbon nitride/porous carbon (g-C_3_N_4_@PC) was synthesized with g-C_3_N_4_ as the precursor and CNCs as the support and was used as a new type of SPME coating. Notably, the newly obtained g-C_3_N_4_@PC coating exhibited superior extraction capabilities compared to a pristine g-C_3_N_4_ coating and commercial coatings, which corresponded to its high surface area and abundant chemical groups. Finally, the proposed analytical method was successfully applied to the determination of trace OCPs in real environmental water and fruit juice samples.

## 2. Experimental

### 2.1. Reagents, Materials, and Instruments

The OCP standards, including etridiazole (ETR), chloroneb (CHE), trifluralin (TRI), hexachlorobenzene (HEX), chlorothalonil (CHT), chlorpyrifos (CHP), alpha-chlordane (α-CHD), and gamma-chlordane (γ-CHD), were provided by AccuStandard Inc. (New Haven, CT, USA). Specific details of the reagents and instruments are clarified in Section S1.

### 2.2. Synthesis of g-C_3_N_4_@PC

Synthesis of g-C_3_N_4_: g-C_3_N_4_ was obtained using high-temperature treatment of urea according to the previous literature. Typically, 15 g urea was placed into a 100 mL crucible and sealed with aluminum foil. Then, the crucible was put into the muffle furnace, the temperature was raised from 30 to 550 °C at a rate of 5 °C min^−1^, and kept for 4 h. Finally, g-C_3_N_4_ powder was obtained after cooling to room temperature.

Synthesis of g-C_3_N_4_@PC: g-C_3_N_4_ powder and CNCs were dispersed in 20 mL of deionized water and subjected to ultrasonication for 2 h to form a suspension. Subsequently, the mixture was put into a freeze dryer and vacuum dried for 2 d to obtain g-C_3_N_4_/CNC aerogels. Then, these yellow aerogels were put into a tube furnace for pyrolysis under N_2_ atmosphere. The temperature of the tubular furnace rose to 550 °C at a rate of 2 °C min^−1^ and kept for 2 h. Finally, the g-C_3_N_4_@PC powder was obtained after cooling to room temperature, where the mass ratios of g-C_3_N_4_ and CNCs were set to 1:1, 2:1, 3:1, 1:2, and 1:3, which were labeled as g-C_3_N_4_@PC-1, g-C_3_N_4_@PC-2, g-C_3_N_4_@PC-3, g-C_3_N_4_@PC-4, and g-C_3_N_4_@PC-5, respectively.

### 2.3. Fabrication of g-C_3_N_4_@PC-Coated SPME Fibers

According to our previous studies [38,39], SPME fibers were fabricated using the adhesive method. Typically, a 3–4 cm long stainless steel wire was washed in nitric acid, sodium hydroxide, and anhydrous ethanol solutions, respectively, and then cleaned with deionized water. After drying, the stainless steel wire was coated evenly with g-C_3_N_4_@PC powder using silicone adhesive. Finally, the g-C_3_N_4_@PC SPME coating was obtained after drying at 130 °C in an oven. It is important to note that the newly acquired g-C_3_N_4_@PC SPME fiber needed to undergo heat treatment at the injection port of GC-MS for 20 min prior to use.

### 2.4. SPME Procedure and GC-MS Analysis

In the SPME process, 10 mL of sample solution was first added into 20 mL of vial. Subsequently, the as-prepared SPME fiber was inserted into the solution for extraction. During the extraction process, the temperature was controlled at 30–70 °C, extraction time was kept at 10–50 min, pH values ranged from 3 to 11, NaCl was used to control the ionic strength of solution from 0 to 30%, and all extraction experiments were repeated 3 times. Upon completion of the extraction, the SPME fiber made was instantly immersed into the GC-MS injection port for detection of the adsorbed substances on the coating. The details of GC-MS parameters and characteristic ion conditions for detecting OCPs are described in Tables S1 and S2.

### 2.5. Analysis of Real Water and Fruit Juice Samples

The real water samples originated from tap water (1#), Weihe River (2#), and rain water (3#), near Henan Normal University (Xinxiang, China). Fruit juice samples, including grape juice (4#), orange juice (5#), and peach juice (6#), which are popular beverages with the public, were purchased from local supermarkets in Xinxiang City, China. To remove any suspended solids and fine impurities from the fruit juice sample, it was centrifuged at 10,000 rpm for 20 min. After that, the resulting supernatant was filtered using a 0.45 μm filter membrane to remove macromolecular substances and minimize the effect of matrix substances. The filtered fruit juices were then diluted in deionized water to a certain proportion. Before analysis, both water and juice samples were stored in a refrigerator at 4 °C.

## 3. Results and Discussion

### 3.1. Characterizations of g-C_3_N_4_@PC Materials

**Figure 1:**
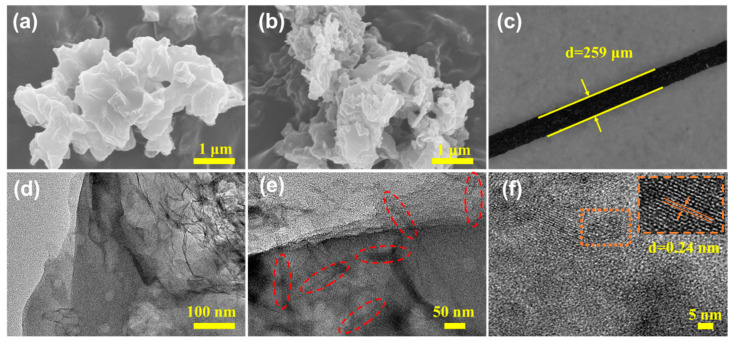
SEM images of (**a**) pure g-C_3_N_4_, (**b**) g-C_3_N_4_@PC-4, (**c**) optical microscope image of as-prepared g-C_3_N_4_@PC-4 SPME fiber and TEM images of (**d**) g-C_3_N_4_, (**e**,**f**) g-C_3_N_4_@PC-4, where, the CNC derived carbon fiber was highlighted with red dotted circle.

The surface morphology and microstructure of the as-prepared g-C_3_N_4_@PC were determined using SEM and TEM (Figure 1). Figure 1a shows that g-C_3_N_4_ presented a disordered porous nanosheet structure. g-C_3_N_4_@PC-4 (Figure 1b) was a nanosheet structure composed of many stacked sheets with a diameter of about 100–200 nm, which showed that under the hydrogen bonding of CNCs, its interlayer connection was more tight, and its layered structure was more clear and irregular. In addition, the optical microscope image of the g-C_3_N_4_@PC-4 SPME fiber showed that the surface of the stainless steel wire was evenly covered by g-C_3_N_4_@PC-4 composites (Figure 1c). The coating thickness was calculated as 64 μm based on the difference between the diameters of the fiber before and after coating, wherein the diameter of the stainless steel wire was 131 μm. Furthermore, TEM analysis (Figure 1d) shows the typical lamellar structure of g-C_3_N_4_. Figure 1e displays the decoration of the CNC-derived carbon fiber onto g-C_3_N_4_. Figure 1f further confirms the synthesis of g-C_3_N_4_@PC material with a lattice distance of 0.24 nm, which is consistent with the (100) crystal plane of the triazine ring unit [40,41].

**Figure 2:**
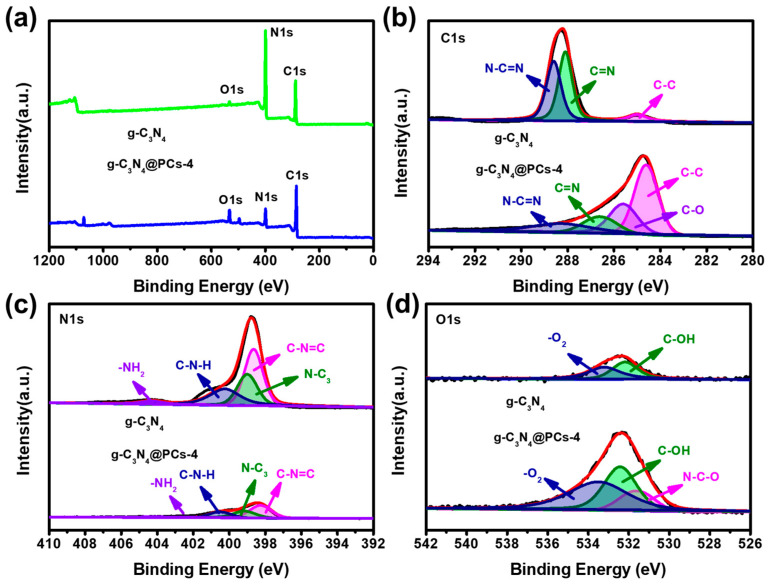
XPS survey spectrum (**a**) and high resolution XPS spectra of C 1s (**b**), N 1s (**c**), and O 1s (**d**) for g-C_3_N_4_ and g-C_3_N_4_@PC-4.

In order to survey the element composition and chemical state of the as-prepared g-C_3_N_4_ and g-C_3_N_4_@PC products, XPS analysis was performed in this study. Figure 2a displays three peaks referring to C 1s (295.63 eV), N 1s (410.63 eV), and O 1s (545.63 eV) both in g-C_3_N_4_ and g-C_3_N_4_@PC. The atomic contents of C, N, O were 55.61%, 42.48%, and 1.9% for g-C_3_N_4_ and 73.57%, 17.94%, and 8.49% for g-C_3_N_4_@PC-4, respectively. The high-resolution spectra of C 1s, N 1s, and O 1s are also shown in Figure 2b–d. For g-C_3_N_4_, it can be observed that the high-resolution spectra of C 1s owned three distinct peaks at 285.1, 288.2, and 288.7 eV, which referred to C–C, C=N, and N–C=N [42,43]. Compared with the g-C_3_N_4_ sample, a new peak was located at 285.8 eV, which corresponded to the C–O groups [44], indicating that the element C in g-C_3_N_4_@PC-4 was partially oxidized. In Figure 2c, the high-resolution N 1s spectra formed four peaks at 398.2, 399.1, 400.9, and 404.6 eV, which belonged to C–N=C, N–C_3_, graphitic N and C–N–H, and –NH_2_, respectively [45]. The O 1s spectra of g-C_3_N_4_ (Figure 2d) displayed two peaks at 532.5 and 533.2 eV, which can be regarded as C–OH and adsorbed O_2_. Meanwhile, a new peak related to N–C–O was located at 531.8 eV in the O 1s spectrum of g-C_3_N_4_@PC-4 [46]. It is worth noting that the intensity of O 1s peak was significantly higher than that of g-C_3_N_4_, which demonstrated that more oxygen-containing functional groups were generated in g-C_3_N_4_@PC-4.

**Figure 3:**
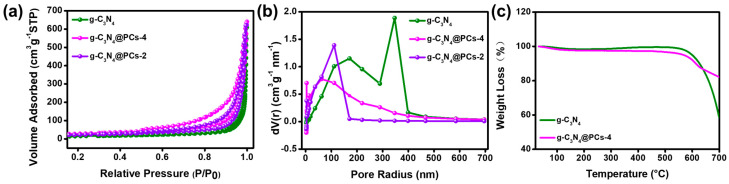
N_2_ adsorption–desorption isotherm (**a**) and BJH pore size distribution (**b**) of as-prepared materials and thermal gravity analysis curve (**c**) of g-C_3_N_4_ and g-C_3_N_4_@PC-4.

To verify the specific surface areas and pore size distributions of the prepared materials, N_2_ adsorption/desorption isotherm curves and the BJH method were performed (Figure 3). The results show that the specific surface areas of g-C_3_N_4_, g-C_3_N_4_@PC-2, and g-C_3_N_4_@PC-4 were 51.1, 79.2, and 98.3 m^2^/g, respectively. According to the BJH method (Figure 3b), the average pore sizes of g-C_3_N_4_, g-C_3_N_4_@PC-2, and g-C_3_N_4_@PC-4 were 85.7, 27.0, and 37.7 nm, respectively, indicating the formation of macroporous structures for g-C_3_N_4_ and mesoporous structures for g-C_3_N_4_@PC. At the same time, the pore volumes of g-C_3_N_4_@PC-4 presented a distinct increase to 0.9784 cm^3^/g compared to pristine g-C_3_N_4_ (0.9145 cm^3^/g). Abundant mesoporous structures and a higher specific surface area and pore capacity of g-C_3_N_4_@PC compared to pristine g-C_3_N_4_ promoted the rapid diffusion of the target analytes and provided more adsorption active sites for the targeted analytes [47]. Moreover, TG was applied to investigate the thermal stability of the composites in this work. Figure 3c demonstrates that the as-prepared composites exhibited excellent thermal stability within 550 °C.

### 3.2. Extraction Performance of g-C_3_N_4_@PC-Coated SPME Fibers

**Figure 4:**
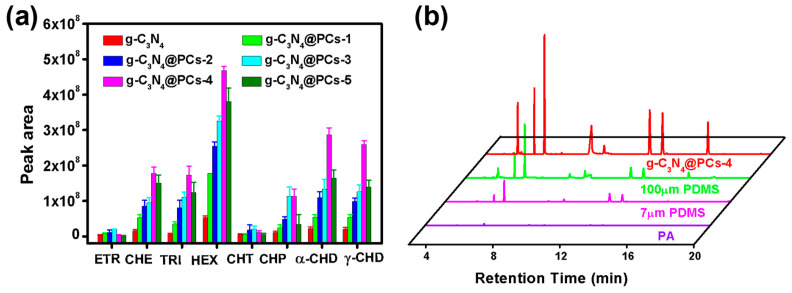
(**a**) Extraction capacities of g-C_3_N_4_@PC with different ratios of g-C_3_N_4_ and CNCs and (**b**) GC-MS chromatograms of OCPs using g-C_3_N_4_@PC-4 and commercial fibers.

To explore the extraction performances of the as-prepared composites, the extraction efficiencies of pristine g-C_3_N_4_ and g-C_3_N_4_@PC toward OCPs were investigated by means of SPME. As shown in Figure 4a, the extraction efficiency of g-C_3_N_4_@PC materials was significantly improved because of higher specific surface areas and richer chemical groups. As the enlargement of CNCs amounts, the extraction efficiencies of g-C_3_N_4_@PC exhibited a trend of first increasing and then decreasing, and g-C_3_N_4_@PC-4 presented the optimum performance, owing to the synergy effects of g-C_3_N_4_ and CNCs. On the one hand, the introduction of CNCs promoted the formation of microporous and mesoporous structures, which increased the pore volume of g-C_3_N_4_@PC, resulting in an improvement in its adsorption capacity for target analytes. On the other hand, there were abundant oxygen-containing functional groups and other chemical groups in g-C_3_N_4_@PC, which promoted adsorption for polar groups. This is consistent with the results of the XPS characterization of g-C_3_N_4_@PC. However, when the amount of CNCs reached the maximum, the extraction efficiency decreased due to the reduction in intermolecular force and the influence of aggregates. Furthermore, comparisons of the extraction efficiencies between the g-C_3_N_4_@PC-4 coating and commercial PA and 7/100 μm PDMS were also studied (Figure 4b). It was found that the extraction efficiencies of g-C_3_N_4_@PC SPME fibers for OCPs were much higher than that of PA and PDMS commercial coatings, with ratios approximately 5–30 and 2–15 times, respectively.

### 3.3. Optimization of g-C_3_N_4_@PC-Coated SPME Conditions

**Figure 5:**
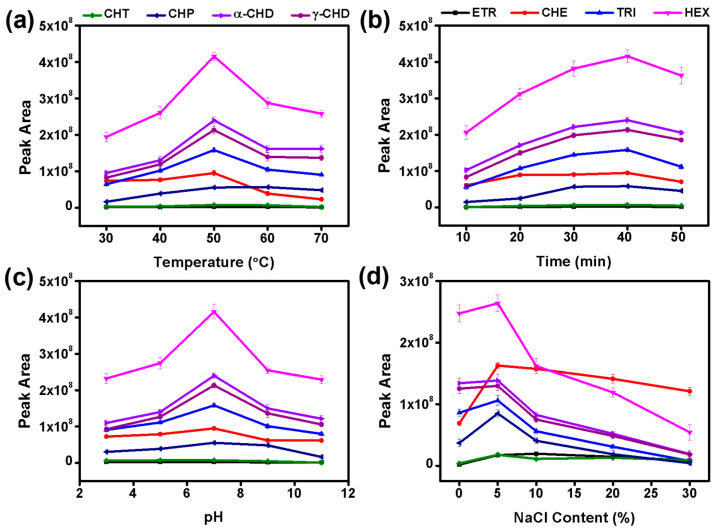
Effect of (**a**) extraction temperature, (**b**) extraction time, (**c**) pH, and (**d**) salt concentration on extraction efficiencies of g-C_3_N_4_@PC SPME coating toward OCPs.

Due to the change in extraction temperature, the distribution coefficient of analytes between the g-C_3_N_4_@PC coating materials and the sample substrate changes and affects the mass and heat transfer effects of extraction efficiency. Increasing the extraction temperature makes for increasing the Henry constant and accelerating the entire extraction process. However, the distribution coefficient between the g-C_3_N_4_@PC coating and the water sample showed a downward trend at this time, which reduces the extraction amount when the system is in equilibrium. Figure 5a displays the influence of temperature on the extraction performance of g-C_3_N_4_@PC for OCPs. The extraction amount of OCPs showed a trend of first increasing and then decreasing as the temperature increased. The optimal extraction temperature for most analytes was 50 °C in this study.

As SPME is a technology related to adsorption equilibrium, the effect of extraction time on extraction performance between 10 and 50 min was also investigated. In Figure 5b, the adsorption capacities for ETR, TRI, HEX, CHT, CHP, α-CHD, and γ-CHD increased with the extension of extraction time and approached the maximum adsorption equilibrium in 40 min. However, the absorption amounts for ETR, TRI, HEX, CHP, and α-CHD gradually showed signs of decreasing with the increase in extraction time, which was due to the potential competitive adsorption effect between OCPs and water [48,49]. In terms of both time consumption and extraction efficiency, the optimal extraction time for this work was set at 40 min.

The impact of solution pH was evaluated from 3 to 11, and the results show that the extraction efficiency reached its maximum at a pH of 7 (Figure 5c). Figure 5d shows the effect of NaCl on the extraction efficiency for OCPs. The results demonstrate that the extraction efficiency increased with a NaCl content of 5%, owing to the salting-out effect, whereas excessive NaCl content in solution reduced the extraction efficiency. This was due to the deposition of NaCl on the coating surface during the high-temperature desorption process in the GC-MS injector, which occupied the adsorption sites, resulting in a diminution in extraction efficiency for OCPs [50].

### 3.4. Method Validation and Real Sample Analysis

#### 3.4.1. Validation of Proposed Analytical Method

**Figure 6:**
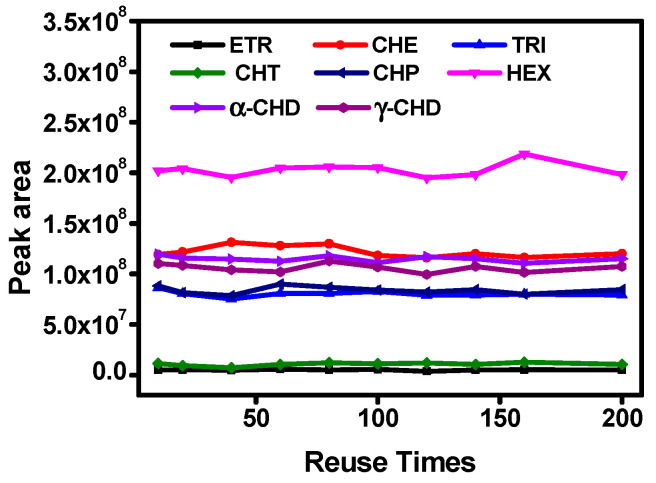
Reusability of g-C_3_N_4_@PC SPME coating toward OCPs.

Under the optimum conditions and parameters, g-C_3_N_4_@PC SPME fibers were used to determine OCPs in water and fruit juice samples coupled with GC-MS. The performance parameters of this method included linear range, detection limit (LOD), limit of quantitation (LOQ), and precision (RSD). The results are listed in Table 1 and Table 2. The proposed method had a linear range of 0.1–1600 ng L^−1^ for water samples with a correlation coefficient above 0.9913 and 0.1–1000 ng L^–1^ for the fruit juice samples. The LOD and LOQ were defined as a signal-to-noise ratio (S/N) of 3 and 10, respectively. The LOD and LOQ for eight OCPs in the water samples are 0.0141–0.0942 ng L^–1^ and 0.0471–0.3140 ng L^–1^; the LOD of the fruit juice samples is 0.0245–0.0777 ng L^–1^, and the LOQ is 0.0819–0.2591 ng L^–1^. The RSD with a single fiber was 5.2–9.3% for water and 4.9–10.5% for fruit juice. The RSD with five fibers was 8.5–10.6% for water and 7.9–10.3% for fruit juice. The proposed method had a wide linear range, good reproducibility, and high sensitivity. To further demonstrate the superiority of the proposed method for determining OCPs, comparisons of the analytical method with other methods that are reported in the literature were investigated. As shown in Table 3, the proposed method presented higher sensitivity. Outstanding performances enabled the proposed method to be successfully applied to the analysis of OCPs in real water and fruit juice samples. Furthermore, we evaluated the reusability of the g-C_3_N_4_@PC SPME coating for determining OCPs. The results (Figure 6) show that there are no distinct changes in extraction efficiency within 200 times of reuse, indicating that the fibers had good durability.

#### 3.4.2. Analysis of Real Water and Fruit Juice Samples

The OCPs in real water samples and fruit juice samples were analyzed using the proposed method. As shown in Table 4 and Table 5, no OCP analytes were found in the tap water (1#), Weihe River (2#), grape juice (4#), and orange juice samples (5#). When the spiked concentration was 2 ng L^–1^, the recovery rate of sample 1# was 93.2–125.5%, the standard recovery rate of sample 2# was 90.6–101.2%, and the recovery rate of sample 3# was 91.4–107.3%. Grape juice (4#) and orange juice (5#) were labeled with 1 ng L^–1^; the recovery rate of sample 4# was 92.3–119.5%, and the recoveries of sample 5# were satisfied with a range of 92.7–113.8%. Peach juice (6#) was labeled with 0.1 ng L^–1^, and the recovery rate of sample 6# was 93.9–106.3%. In summary, the established method of combining the coating material with a GC-MS instrument obtained a satisfactory recovery rate for determining OCPs both in water and fruit juice samples.

## 4. Conclusions

In this study, a novel g-C_3_N_4_@PC composite was synthesized using CNCs as the support. In view of its outstanding advantages of a high specific surface area, porous structure, and abundant chemical groups, the g-C_3_N_4_@PC composite was used as an SPME coating for extracting OCPs. The mesoporous channels in g-C_3_N_4_@PC were helpful to enhance the mass transfer rate of the analytes. In addition, the addition of CNCs made g-C_3_N_4_ have a rich pore structure, providing more active sites for the adsorption and transportation of analytes; therefore, the g-C_3_N_4_@PC-coated fiber exhibited higher extraction efficiency for OCPs than pristine g-C_3_N_4_ and commercial SPME coatings. The established method was successfully applied to the analysis of OCPs in environmental water samples and fruit juice samples. This study proposed a novel SPME coating material for capturing and determining trace organic pollutants from environmental media and food samples.

## Figures and Tables

**Table 1:** Extraction capacities of g-C_3_N_4_@PC SPME fibers toward OCPs from water.

Analytes	Linear Ranges (ng L^−1^)	R^2^	LOD (ng L^−1^)	LOQ (ng L^−1^)	RSD (%)	
					One Fiber (*n* = 7)	Fiber-to-Fiber (*n* = 5)
ETR	0.1–1600	0.9995	0.0224	0.0749	5.2	8.9
CHN	0.1–1600	0.9960	0.0942	0.3140	7.3	9.2
TRI	0.1–1600	0.9913	0.0706	0.2355	6.4	8.5
HCB	0.1–1600	0.9972	0.0565	0.1884	8.9	10.7
CHT	0.1–1600	0.9978	0.0668	0.2228	7.6	9.4
CHP	0.1–1600	0.9960	0.0141	0.0471	9.3	9.7
α-CHD	0.1–1600	0.9933	0.0471	0.1570	6.5	10.1
γ-CHD	0.1–1600	0.9917	0.0565	0.1884	5.7	10.6

**Table 2:** Analytical performance of g-C_3_N_4_@PC SPME fibers toward OCPs from fruit juices.

Analytes	Linear Ranges (ng L^−1^)	R^2^	LOD (ng L^−1^)	LOQ (ng L^−1^)	RSD (%)	
					One Fiber (*n* = 7)	Fiber-to-Fiber (*n* = 5)
ETR	0.1–1000	0.9924	0.0777	0.2591	4.9	7.9
CHN	0.1–500	0.9930	0.0282	0.0942	7.6	8.2
TRI	0.1–500	0.9904	0.0403	0.1345	5.4	9.5
HCB	0.1–500	0.9929	0.0256	0.0856	7.8	8.7
CHT	0.1–1000	0.9982	0.0565	0.1884	8.4	9.6
CHP	0.1–1000	0.9994	0.0314	0.1046	9.6	8.7
α-CHD	0.1–1000	0.9993	0.0245	0.0819	10.5	10.3
γ-CHD	0.1–1000	0.9967	0.0282	0.0941	6.7	10.1

**Table 3:** The comparison of methods for determination of OCPs in water.

Analytical Methods	Coating Materials	Linear Ranges (ng L^–1^)	LOD (ng L^–1^)	LOQ (ng L^–1^)	Reference
SPME/GC-MS	mesoporous TiO_2_	5–5000	0.08–0.60	0.27–2.00	[50]
HS-SPME/GC-ECD	porous aromatic framework/ionic liquid	1000–500,000	110–290	350–930	[51]
SPME/GC-MS	sol–gel–graphene	10–100,000	0.19–18.30	–	[52]
HS-SPME/GC-MS	DVB/CAR/PDMS	20–100,000	20–230	20–770	[53]
SPE/GC-ECD	Au/Ti-UVM-7	400–100,000	0.30–20	1–61	[54]
SPME/GC-ECD	AuNPs	560–10,000	130–240	440–810	[55]
SPME/GC-MS	carbonized polydopamine	10–50,000	1.40–15	–	[56]
SPME/GC-MS	C_18_ composite	2–500	0.059–0.151	–	[57]
SPME/GC-MS	g-C_3_N_4_@PC	0.1–1600	0.0141–0.0942	0.0471–0.3140	This work

**Table 4:** Analytical results and recoveries for determination of OCPs from real water samples.

Analytes	Tap Water (1#)			Weihe River (2#)			Rain Water (3#)		
	Found (ng L^–1^)	RSD (%, *n* = 3)	Recoveries (%, Spiked with 2 ng L^–1^)	Found (ng L^–1^)	RSD (%, *n* = 3)	Recoveries (%, Spiked with 2 ng L^–1^)	Found (ng L^–1^)	RSD (%, *n* = 3)	Recoveries (%, Spiked with 0.2 ng L^–1^)
ETR	ND	–	125.5	ND	–	94.1	ND	–	93.5
CHN	ND	–	104.8	ND	–	93.4	ND	–	107.3
TRI	ND	–	96.4	ND	–	90.6	10.9	–	94.1
HCB	ND	–	122.4	ND	–	93.9	ND	–	95.7
CHT	ND	–	103.3	ND	–	101.2	ND	–	98.8
CHP	ND	–	93.2	ND	–	95.4	ND	–	93.3
α-CHD	ND	–	98.1	ND	–	98.3	ND	–	102.7
γ-CHD	ND	–	95.6	ND	–	97.6	ND	–	91.4

ND represents not detected.

**Table 5:** Analytical results and recoveries for determination of OCPs from fruit juice samples.

Analytes	Grape Juice(4#)			Orange Juice(5#)			Peach Juice(6#)		
	Found (ng L^–1^)	RSD (%, *n* = 3)	Recoveries (%, Spiked with 1 ng L^–1^)	Found (ng L^–1^)	RSD (%, *n* = 3)	Recoveries (%, Spiked with 1 ng L^–1^)	Found (ng L^–1^)	RSD (%, *n* = 3)	Recoveries (%, Spiked with 0.1 ng L^–1^)
ETR	ND	–	92.3	ND	–	92.7	ND	–	96.8
CHN	ND	–	94.4	ND	–	95.2	ND	–	97.2
TRI	ND	–	97.3	ND	–	98.2	10.9	–	95.6
HCB	ND	–	94.8	ND	–	95.6	ND	–	106.3
CHT	ND	–	107.9	ND	–	113.8	ND	–	98.2
CHP	ND	–	119.5	ND	–	94.3	ND	–	93.9
α-CHD	ND	–	95.8	ND	–	92.6	ND	–	96.1
γ-CHD	ND	–	98.7	ND	–	108.3	ND	–	95.5

ND represents not detected.

## Data Availability

This study presents novel concepts and did not report any data.

## Supplementary Materials

The following supporting information can be downloaded at: https://www.mdpi.com/article/10.3390/polym15092218/s1, Section S1: Apparatus used for characterization of materials; Table S1: Operating parameters of GC–MS; Table S2: Retention time and characteristic ions of OCPs.
