# Supplementary material for: Cellulose Nanocrystals Induced Loose and Porous Graphite Phase Carbon Nitride/Porous Carbon Composites for Capturing and Determining of Organochlorine Pesticides from Water and Fruit Juice by Solid-Phase Microextraction
Source: Polymers (Basel). 2023 May 8;15(9):2218. doi: 10.3390/polym15092218 (PMC10181374; doi:10.3390/polym15092218)
Supplement: Supplementary file 1 [file polymers-15-02218-s001.zip › polymers-2329612-supplementary.pdf]

Supplementary Information

Cellulose Nanocrystals Induced Loose and Porous Graphite Phase Carbon Nitride/Porous Carbon Composites for Capturing and Determining of Organochlorine Pesticides from Water and Fruit Juice by Solid-Phase Microextraction

Huimin Li [1], Panlong Dong [1], Anying Long [2,*], Suling Feng [1], Jing Fan [1] and Shengrui Xu [1,*]

[1] Key Laboratory of Green Chemical Media and Reactions, Ministry of Education, Collaborative Innovation Center of Henan Province for Green Manufacturing of Fine Chemicals, School of Environment, School of Chemistry and Chemical Engineering, Henan Normal University, Xinxiang 453007, China; ming1391027849@163.com (H.L.); pldong2000@163.com (P.D.); slfeng@htu.cn (S.F.); fanjing@htu.cn (J.F.)

[2] 113 Geological Brigade, Bureau of Geology and Mineral Exploration and Development Guizhou Province, Liupanshui 553000, China

* Correspondence: longying031042@163.com (A.L.); xushengrui@126.com (S.X.)

Section S1. Characterization material equipment and laboratory reagents

Sodium chloride (NaCl), sodium hydroxide (NaOH), and urea ($CO(NH_2)_2$) were obtained from Damao Chemical Reagent Factory (Tianjin, China). CNC was bought from Shansi Nanotechnology Co., Ltd. (Shanghai, China). 100 μm stainless-steel wire was bought from Shenzhen Hubei Baofeng Industrial Co., Ltd. (Shenzhen, China). Sylard184 silicone elastomer was purchased by Dow Silicones Corporation (Seneffe, Belgium, USA). The three commercial SPME fibers, namely (100/7 μm) PDMS and PA were obtained from ANPEL Laboratory Technologies Inc. (Shanghai, China).

The microstructures of the g-C_3N_4@PCs were characterized by a scanning electron microscope (SEM, SU8010, Hitachi, Tokyo, Japan) and a transmission electron microscope (TEM, JEM2100, JEOL Electronics, Japan). X-ray diffraction spectra (XRD, X'Pert3 Powder, PANalytical B.V., Almelo, Holland) was used to characterize the compositions of g-C_3N_4@PCs. Fourier-transform infrared (FTIR) spectra were recorded by a Thermo Nicolet NEXUS spectrometer (Thermo Fisher Scientific, Waltham, MA, USA) in the range of 4000–400 cm^{-1}. The surface chemical groups were detected by X-ray photoelectron spectroscopy (XPS, VG Multilab 2000 X spectrometer, Thermo Fisher Scientific, Waltham, MA, USA). Analyze the pyrolysis temperature of g-C_3N_4@PCs materials by comprehensive thermal analyzer (TG, STA449C, NETZSCH, Germany). The BET surface area and pore size distribution were obtained by a nitrogen adsorption/desorption apparatus (Micromeritics ASAP 2020 M, Atlanta, GA, USA). At the same time, GC-MS (Agilent 7890B and 7000D, Santa Clara, California, USA) was applied to the accurate detection of OCPs.

Table S1. Operating parameters of of GC-MS.

GC-MS injector	280 °C with splitless mode
**Column type**	HP-5 MS (30 m × 0.25 mm × 0.25 μm)
**Flow rate of column**	1 mL min^{-1}
**Column oven**	The initial temperature is 80 °C, and the temperature rises to 200 °C at the rate of 30 °C/min for 8

	min; then rise to 230 °C at 5 °C/min for 2 min.
**MS Quad temperature**	150 °C
**MS source temperature**	230 °C

Table S2. Retention time and characteristic ions of OPCs

Compound	Reference ion	Quantitative ion	Retention time / min
ETR	213、211、185、183	211	4.669
CHE	208、206、193、191	191	4.939
TRI	306、264	306	5.941
HEX	286、284、282	284	6.556
CHT	267、266、264	266	7.615
CHP	303、301、249	301	10.199
α –CHD	377、375、373、371	373	12.954
γ –CHD	377、373、374、371	373	13.708
